# Global decline in vaccine coverage; COVID-19 Vaccine Hesitancy: Ulrich Laaser

**DOI:** 10.1093/eurpub/ckac129.735

**Published:** 2022-10-25

**Authors:** U Laaser

**Affiliations:** Bielefeld School of Public Health, Bielefeld, Germany

## Abstract

Vaccine hesitancy is a relatively new concept, developed by WHO's Strategic Advisory Group of Experts (SAGE) on Immunization in 2014 as a response to the growing awareness of the decline in global confidence in vaccination. Vaccine hesitancy is a context-specific behavioural phenomenon whose occurrence ranges between full acceptance and complete refusal of vaccines. Several studies have explored factors that influence people's decision to get vaccinated and in 2018 WHO and UNICEF conducted a joint study to explore the reasons for vaccine hesitancy. The study aimed to determine the reported rate of vaccine hesitancy across the globe and the reasons for hesitancy. In most studies three top reasons were identified. 1) vaccine safety concerns, 2) lack of knowledge and awareness of vaccine importance, and 3) religion, culture, gender and socio-economic issues regarding vaccines. Other factors contributing are negative perception of vaccine efficacy, safety, convenience, and price. Some of the consistent socio-demographic groups that were identified to be associated with increased hesitancy included: women, younger participants, and people who were less educated, had lower income, had no insurance, lived in a rural area, and self-identified as a racial/ethnic minority. Vaccine hesitancy is associated with the global crisis of trust in science and institutions, namely lack of political trust, which can be defined as public judgment that the system and its representatives are responsive and reliable. Furthermore, distrust in one institution is related to distrust in other, indicating the unidimensional phenomenon. This kind of distrust is exemplified by the appearance of infodemic - an overabundance of information. In addition, support for conspiracy theories related to COVID-19 which correlates with the scepticism towards vaccination has significantly higher rates among Balkans’ populations.

